# Flowering plant immune repertoires expand under mycorrhizal symbiosis

**DOI:** 10.1002/pld3.125

**Published:** 2019-03-05

**Authors:** Eric M. Kramer, Samantha A. Statter, Ho Jun Yi, Joseph W. Carlson, Donald H. R. McClelland

**Affiliations:** ^1^ Department of Physics Bard College at Simon's Rock Great Barrington Massachusetts; ^2^ Lawrence Berkeley National Laboratory Joint Genome Institute Berkeley California; ^3^ Department of Environmental Science Bard College at Simon's Rock Great Barrington Massachusetts

**Keywords:** arbuscular mycorrhiza, disease‐resistance genes, ectomycorrhiza, R‐genes, rhizobia, symbiosis

## Abstract

Immune perception in flowering plants is mediated by a repertoire of cytoplasmic and cell‐surface receptors that detect invading microbes and their effects on cells. Here, we show that several large families of immune receptors exhibit size variations related to a plant's competence to host symbiotic root fungi (mycorrhiza). Plants that do not participate in mycorrhizal associations have significantly smaller immune repertoires, while the most promiscuous symbiotic hosts (ectomycorrhizal plant species) have significantly larger immune repertoires. By contrast, we find no significant increase in immune repertoire size among legumes competent to form a symbiosis with nitrogen‐fixing bacteria (rhizobia). To explain these observations, we hypothesize that plant immune repertoire size expands with symbiote species diversity.

## 1 INTRODUCTION

Fungi colonized land before plants, and plant‐fungi symbiosis was a key step in the evolution of land plants (Delaux et al., 2015; Lutzoni et al., 2018; Wang et al., 2010). Thus, early lineages of terrestrial plants already had a molecular toolkit for fungal symbiosis, and over time these associations diversified to include a wide range of fungal and bacterial species.

Plant‐rhizobia symbiosis is the best‐studied example, as legume crops are important for human nutrition (reviewed in Dilworth, James, Sprent, & Newton, 2008). Rhizobial host plants have evolved signaling pathways that allow the bacteria to enter and colonize the root without triggering a sustained defense response (Kouchi et al., 2004; Lohar et al., 2006; Maunoury et al., 2010). Once inside target root cells, rhizobia stimulate the development of specialized plant organs called nodules that facilitate the exchange of rhizobial nitrogen for plant host‐derived carbon compounds.

Although less widely studied than rhizobial symbiosis, symbioses between plants and fungi are older, more diverse, and much more widespread (Wang et al., 2010; Delaux et al., 2015; Lutzoni et al., 2018; reviewed in Smith & Read, 2008). There are thousands of species of mycorrhizal fungi, and in their various forms they play an important yet still incompletely understood role in plant nutrition. Typically, the fungal hyphae form a continuum from the soil into the outer layers of the root, effectively expanding the soil volume explored by the root system and accumulating critical elements like phosphorus for use by the plant.

In this paper, we will focus on two categories of mycorrhizal associations: arbuscular mycorrhiza and ectomycorrhiza. The association between arbuscular mycorrhiza (AM) and plants is ubiquitous, with 80% of terrestrial plants competent to form these symbioses (reviewed in Wang & Qiu, 2006; Smith & Read, 2008). Morphologically, AM are distinguishable from other mycorrhizal fungi by the presence of densely coiled or ramified hyphae located inside living cells of the root cortex. AM are named after these ramified hyphae, which are called “arbuscles.” Ectomycorrhizal (ECM) symbioses are more recently evolved and much less common among plant species (3%), but they are especially important for forest trees (Smith & Read, 2008). The hyphae of ECM fungi form a network that densely occupies the spaces between the cells of the outer plant root, but they do not penetrate living host cells.

An additional category of plants comprises those species that have lost their competence for mycorrhizal symbiosis (reviewed in Wang & Qiu, 2006; Brundrett, 2017). Nonmycorrhizal species (NM) have evolved multiple times from AM species. They are generally short‐lived, non‐rhizobial, and nonwoody. Notably, NM species include the model species *Arabidopsis thaliana* and other members of the Brassicaceae.

In this paper, we examine the possible connection between plant symbiotic competence and the innate immune repertoire.

Plants lack mobile defensive cells, so their resistance to microbial pathogens is mediated by an innate immune response (reviewed in Chisholm, Coaker, Day, & Staskawicz, 2006; Jones & Dangl, 2006; Bent & Mackey, 2007; Cook, Mesarich, & Thomma, 2015; Hacquard, Spaepen, Garrido‐Oter, & Schulze‐Lefert, 2017). Pathogen perception can take place outside the cell or in the cytoplasm. Outside the cell, transmembrane receptor kinases are sensitive to pathogen‐associated or damage‐associated molecular patterns (Brutus, Sicilia, Macone, Cervone, & De Lorenzo, 2010; Gomez‐Gomez & Boller, 2000; Miya et al., 2007). These receptor kinases signal to downstream targets, eventually triggering a range of immune responses from the plant. A second signaling pathway involves events in the cytoplasm. To evade the host immune response, pathogens secrete effector proteins that interfere with immune signaling or downstream events (reviewed in Lo Presti et al., 2015). Host proteins in the Nucleotide Binding Site family (NBS, also known as NB‐ARC) monitor the cytoplasm for these effectors, and probably also for changes in effector targets, and thereby provide a second mode of pathogen detection (Botella et al., 1998; Collins et al., 1999; Wang et al., 1999). The majority of disease‐resistance loci (R‐genes) discovered in plants map onto genes with NBS domains, and the term “R‐genes” is sometimes used synonymously with NBS genes (reviewed in Sekhwal et al., 2015). A third mechanism for plant‐pathogen interactions involves the exchange of small RNAs evolved to silence plant immune responses or microbe pathogenicity, respectively (Cai et al., 2018; Weiberg et al., 2013).

While the traditional view is that the immune system evolved to defend the plant against pathogens, there is a growing list of cases in which immune signaling pathways also mediate plant interactions with nonpathogenic microbes. One important example is the LysM family of receptor kinases, which includes receptors that perceive the fungal cell wall component chitin. In rice, this family includes OsCERK1, which has a role in both fungal pathogen response and the establishment of AM symbiosis (Miyata et al., 2014). In legume‐rhizobia symbiosis, some LysM receptors perceive chitin‐like signals called Nod factors produced by symbiotic bacteria (Madsen et al., 2003). There is also some evidence that the NBS family has a role in symbiosis. NBS genes have been shown to regulate host‐rhizobia specificity in some legumes (Yang, Tang, Gao, Krishnan, & Zhu, 2010). These and other examples have prompted recent reviews (Cook et al., 2015; Hacquard et al., 2017) to describe the plant immune system as a “microbe management” or “surveillance” system, rather than a defense system per se.

In this paper, we initially focus on the NBS gene family. The NBS gene family is remarkable for both its overall size – often comprising several percent of the entire genome – and for the wide size variation between species. Counts of NBS range from ~40 in eelgrass (*Zostera marina*) to ~1,000 in apple (*Malus domestica*) (Olsen et al., 2016; Velasco et al., 2010). Beginning with the sequencing of the first tree genome, *Populus trichocarpa* (black cottonwood), several authors observed that woody perennials, especially trees, have relatively large NBS families (see Tuskan et al., 2006; Yang, Zhang, Yue, Tian, & Chen, 2008 and the opinion article Tobias & Guest, 2014). More recently, Plomion et al. (2018) compared the genomes of nine woody and seven nonwoody species and found several large clades of NBS genes that show significant expansions in woody species. They speculated that the expansion of defense‐related gene families is related to the long life‐span of woody perennials.

In work preliminary to this paper, we saw hints of an alternative explanation for variations in NBS family size. We noted that species with small NBS families tend to be NM. We also noticed that tree species with especially large NBS families were typically ECM. We thus set out to establish the statistical significance of this result, to find other gene families with similar correlations, and to seek possible explanations in the ecology and evolution of flowering plants.

## 2 METHODS

### 2.1 Genomes

We downloaded the genomes of 39 species of angiosperms, including 9 monocots (2 NM and 7 AM) and 30 eudicots (5 NM, 20 AM, and 5 ECM). Of the 39 genomes, 38 are public. Permission to use the 39th, *Salix purpurea*, was generously granted prior to the end of the official embargo. A complete list of sources is provided in the Supporting Information Table S1. The majority were downloaded from the Phytozome archive ( https://phytozome.jgi.doe.gov; Goodstein et al., 2012).

To provide consistency across genomes, we limited consideration to one transcript per locus, called the “primary transcript” in Phytozome annotations. This was to avoid statistical bias due to large differences in the completeness of various genome annotations. For example, Arabidopsis TAIR10 averages 1.29 transcripts per locus, while many species genomes are still in version 1.0 and are annotated with exactly one transcript per locus.

### 2.2 Protein domains

Pfam domains were identified using PfamScan (Punta et al., 2012) and transmembrane domains were identified using Phobius (Kall, Krogh, & Sonnhammer, 2007).

### 2.3 Species data

**Figure 1:**
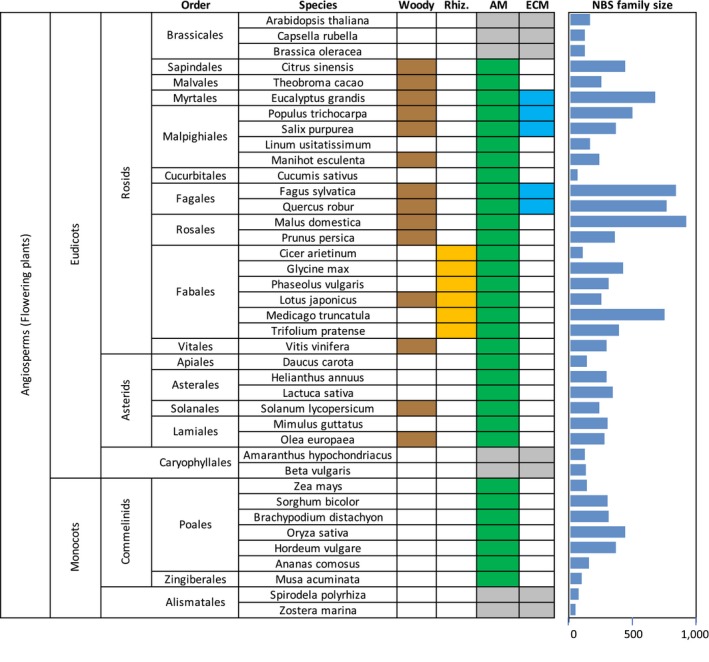
Summary of the 39 species of angiosperms used in this paper. Taxonomy shown on the left follows Ref. (APGIV 2016). In the color‐coded boxes, brown = woody perennial, orange = rhizobial species, gray = nonmycorrhizal (NM) species, green = arbuscular mycorrhizal (AM) species, light blue = ectomycorrhizal (ECM) species. Bars on the right show the size of the NBS gene family in each species, as counted by PfamScan (Punta et al., 2012). References for mycorrhizal status may be found in Supporting Information Table S1

We collected the following information for each species: mycorrhizal and rhizobial symbiotic competence, annual/perennial life cycle, and woodiness (i.e. capable of secondary growth and lignification). This information was collected from a variety of sources, as detailed in the Supporting Information. We were careful to include only species whose mycorrhizal status has been confirmed by published observation. Figure 1 summarizes some properties of the 39 species used, including a taxonomy after (APGIV 2016). The woody category includes trees and vines having woody secondary growth. It also includes *Lotus japonicus,* which has nonhardy stems but a perennial woody taproot, and *Solanum lycopersicum*, which, while typically grown as an annual crop, develops a woody stem and roots when grown as a perennial (Peralta, Spooner, & Knapp, 2008).

### 2.4 Family expansion

To test whether a gene family expands between two sets of species, we calculated a *p*‐value using the exact, one‐sided Wilcoxon‐Mann‐Whitney rank sum test, implemented using the COIN package in R (COIN version 1.2‐2, Hothorn, Hornik, van de Wiel, & Zeileis, 2006; R version 3.5.1, Team, 2018). In cases with multiple simultaneous tests, the technique of Benjamini and Hochberg (1995) was used to limit the false discovery rate (FDR) to <5%.

### 2.5 Linear regression

We used Excel (v.16, Microsoft) to do a simultaneous regression of family size on woodiness and mycorrhizal competence using the equation

$$n_{i}=a+b_{w}x_{\mathrm{iw}}+b_{\mathrm{myc}}x_{i,\mathrm{myc}}$$

where *i* is an index that ranges over the 39 species in our data set, *n*_*i*_ is the number of loci in the gene family of interest in species *i*,* a*,* b*_w_, and *b*_myc_ are the three coefficients to be determined, *x*_iw_ is assigned a value of −1 if species *i* is nonwoody and +1 if woody, and *x*_i,myc_ is assigned a value of −1 if species *i* is NM, 0 if AM, and +1 if ECM. Note that *x*_iw_ and *x*_i,myc_ are defined to have identical ranges (from −1 to +1) so the relative size of the regression coefficients provides a measure of the relative importance of woodiness and mycorrhizal competence. We tested whether each regression coefficient was significantly different from 0 using a *t* test (Excel v.16, Microsoft).

### 2.6 Immune response

To assess whether a gene family is over‐represented in the immune response, we used transcriptome data from the following papers. Wan et al. (2008) applied 1 μM chitooctoase (chitin, a ubiquitous component of fungal cell walls) to *A. thaliana* seedlings 14 days after germination (DAG) grown in liquid media. They used an Affymetrix ATH‐1 gene chip to identify 663 genes up‐regulated after 30 min. Denoux et al. (2008) applied 50 μg/ml oligogalacturonides (OGs, an immune elicitor derived from the breakdown of plant cell walls) degree of polymerization 9–16, to 10 DAG *A. thaliana* seedlings grown in liquid media. In a separate experiment, they applied 1 μM Flg22, a peptide sequence derived from bacterial flagellin. In these cases, they reported 990 and 1,437, respectively, up‐regulated genes after 1 hr. Bernsdorff et al. (2016) induced systemic acquired resistance (SAR) by inoculating the lower leaves of mature Arabidopsis plants with the bacterial pathogen *Pseudomonas syringae*, waiting 48 hr, then collecting uninoculated leaves from higher up the stem. They found 3,413 genes up‐regulated as compared with control leaves. Foster, Pelletier, Tanguay, and Seguin (2015) sprayed spores of a fungal pathogen (genus *Sphaerulina*) on 6‐month‐old *Populus tremuloides* seedlings. RNA‐seq was used to identify differentially regulated genes in mature leaves at 1, 4, and 15 days after inoculation (dai). The number of up‐regulated loci was 276, 1,712, and 5,249 at 1, 4, and 15 dai, respectively.

Over‐representation was assessed using a hypergeometric test to find the *p*‐value (Excel v.16, Microsoft), then further restricted to FDR < 5% (Benjamini & Hochberg, 1995).

### 2.7 AM‐related gene lists

To assess whether a gene family is over‐represented in lists related to AM competence, we used data from the following papers. Delaux et al. (2014) compared genomic and transcriptomic data from ~40 plant species to identify a list of 174 *Medicago truncatula* genes conserved in AM‐competent species but lost in NM species. Bravo, York, Pumplin, Mueller, and Harrison (2016) conducted a similar phylogenomic analysis to identify a list of 138 genes. Sugimura and Saito (2017) used RNA‐seq to compare the root transcriptomes of 4‐week‐old *S. lycopersicum* plants grown with or without an inoculation of spores from the AM fungal species *Rhizophagus irregularis*. They found 744 genes up‐regulated in the AM‐treated plants. Recchia, Konzen, Cassieri, Caldas, and Tsai (2018) used RNA‐seq to compare root transcriptomes of *Phaseolus vulgaris* plants grown with or without AM fungal inoculum. They found 714 genes up‐regulated in the plants grown with AM. Vangelisti et al. (2018) used RNA‐seq to determine the transcriptomes of *Helianthus annuus* seedling roots 16 days after inoculation with the AM fungal species *Rhizoglomus irregulare*. Compared to controls without the inoculation, they found 694 genes up‐regulated in AM plants.

Over‐representation was assessed using a hypergeometric test to find the *p*‐value (Excel v.16, Microsoft), then further restricted to FDR < 5% (Benjamini & Hochberg, 1995).

## 3 RESULTS

### 3.1 NBS family size depends on mycorrhizal competence

**Figure 2:**
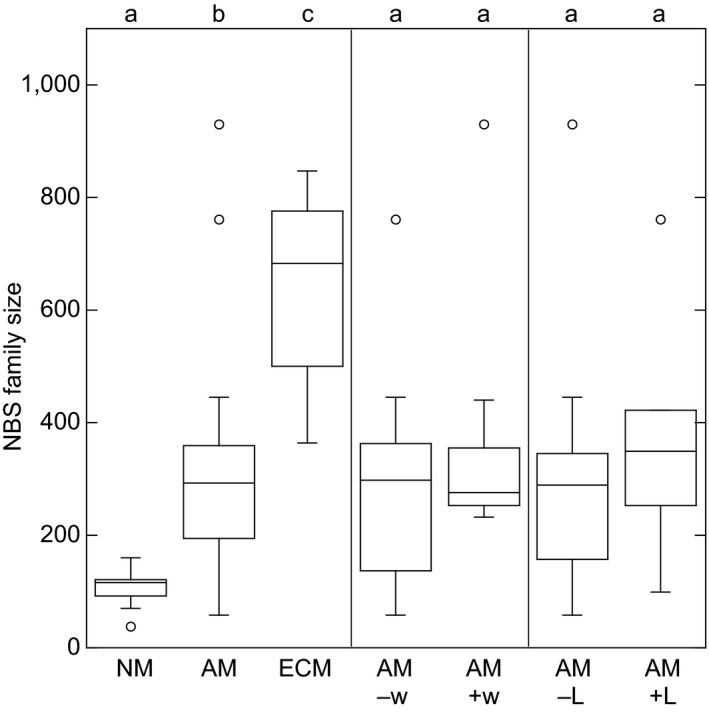
Size distribution of the NBS gene family. Left panel: NBS family size in nonmycorrhizal (NM), arbuscular mycorrhizal (AM), and ectomycrorrhizal (ECM) plant species (*n *=* *7, 27, and 5, respectively). Center panel: NBS family size in nonwoody AM species (−w) and woody AM species (+w) (*n *=* *18 and 9, respectively). Right panel: NBS family size in non‐rhizobial AM species (−L) and rhizobial AM species (+L) (*n *=* *21 and 6, respectively). Letters at top of panel indicate significantly different cohorts (Wilcoxon‐Mann‐Whitney rank sum test, *p* < 0.05; within‐panel comparisons only)

We determined the size of the NBS gene family in 39 species of angiosperms for which mycorrhizal status was also available (Figure 1, Supporting Information Tables S1, S2). Figure 2 shows the distribution of NBS family size in host species with different mycorrhizal competence. The median number of NBS loci in NM, AM, and ECM species is 116, 293, and 683, respectively. Comparing these groups using nonparametric statistics, we find that NM plant species have significantly smaller NBS families than AM species (*p* = 3e‐4) and ECM plant species have significantly larger NBS families than either NM or AM species (*p* = 1e‐3 for both comparisons).

Note that all seven NM species in our list are nonwoody while the five ECM species are woody perennials (Figure 1). Thus, we chose to examine the relative importance of woodiness and mycorrhizal competence for NBS family size using a simultaneous linear regression (results in Supporting Information Table S3). The regression coefficient associated with woodiness was positive, but not significantly different from 0 (*p* = 0.10 using a *t*‐test). The regression coefficient associated with mycorrhizal competence was about 5× larger, and was significantly different from 0 (*p* = 6e‐4 using a *t*‐test).

We also tested for a dependence on woodiness using the subset of 27 AM species. The AM‐competent species were divided into two cohorts of 18 nonwoody species and 9 woody species, respectively (Figure 1). The distributions of NBS family sizes are compared in Figure 2. Median NBS counts of the nonwoody and woody AM‐competent species are 298 and 276, respectively, and nonparametric statistics find no significant difference between the two groups (*p* = 0.13 using an exact, one‐sided Wilcoxon‐Mann‐Whitney rank sum test).

Since mycorrhizal competence has such a large effect on NBS family size, we also tested the possible effect of symbiosis with rhizobia. We divided our AM‐competent species into two cohorts of 21 nonrhizobial and 6 rhizobial plant species, respectively (Figure 1). While the distributions shown in Figure 2 suggest a small NBS expansion in the rhizobia‐competent species, nonparametric statistics finds no significant difference between the two sets (*p* = 0.29 using an exact, one‐sided Wilcoxon‐Mann‐Whitney rank sum test).

### 3.2 Several other gene families also expand with mycorrhizal competence

We next searched for other gene families with properties similar to NBS, namely large size and a correlation with mycorrhizal competence. We compiled a list of gene families using the Pfam annotation of protein domains (Punta et al., 2012). A Pfam domain was categorized as large if the average number of loci per species was 20 or greater (the cutoff of 20 is arbitrary). Because preliminary work had indicated the likely importance of the kinase and leucine‐rich repeat (LRR) superfamilies, and because these superfamilies show substantial overlap, the relevant Pfam domains were curated into more commonly used categories: LRR‐receptor‐like kinases (LRR‐RLKs), receptor‐like proteins (RLPs), and soluble kinases. The resulting list of large gene families contains 331 entries (Supporting Information Table S2).

**Figure 3:**
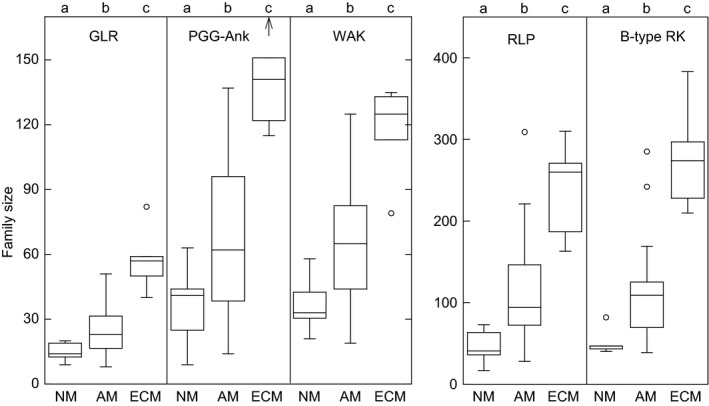
Size distribution of mycorrhizal‐expanded (MycEx) gene families. GLR = glutamate receptor‐like proteins, PGG‐Ank = PGG‐ankyrins, WAK = wall‐associated receptor kinases, RLP = receptor‐like proteins, B‐type RK = bulb‐type lectin receptor kinases. Mycorrhizal competence abbreviations same as Figure 1 (*n *=* *7, 27, and 5 for NM, AM, and ECM, respectively). Letters at top of panel indicate significantly different cohorts (Wilcoxon‐Mann‐Whitney rank sum test, false discovery rate < 0.05; within‐panel comparisons only, e.g. GLR and WAK are not compared to each other)

For each gene category, we used nonparametric statistics to calculate a *p*‐value characterizing gene loss in NM vs AM species, and gene gains in AM vs ECM species. In both comparisons, we limited the false discovery rate to 5% using the technique of Benjamini and Hochberg (1995). There are 22 gene categories that show significant differences in both comparisons (category 1 in Supporting Information Table S4). We next required the category size differences to be large, with at least a 50% gain from NM to AM, and again from AM to ECM. The final list is readily curated into just six families (the three Pfam domains PF01453, PF00954, and PF08276 occur together in members of the Bulb‐type lectin receptor kinase family). These include the NBS family, two families of receptor‐like kinases, the glutamate receptor‐like family (GLR), a large clade of ankyrins (PGG‐ankyrins), and the receptor‐like proteins (RLP). We call these the Mycorrhizal‐Expanded (MycEx) gene families (Figure 3, Supporting Information Tables S5 and S6).

Starting with the same list of 331 gene categories, we also looked for gene family expansions in woody vs nonwoody AM species, and for families that expand in the rhizobial legumes as compared with other AM species. In both cases, no significant family expansions were found.

### 3.3 Properties of the MycEx gene families

The MycEx gene families are prominent in several immune response transcriptomes. In *A. thaliana*, a nonmycorrhizal model species, all six families were over‐represented in the response to *P. syringae* infection, and the majority were over‐represented in response to the immune elicitors flagellin, chitin, and oligogalacturonides (Bernsdorff et al., 2016; Denoux et al., 2008; Wan et al., 2008) (Supporting Information Table S7, FDR < 5%). The transcriptional response of *P. tremuloides* (quaking aspen) leaves to a fungal pathgogen shows a phase at 15 days after inoculation (dai) where all MycEx families except PGG‐ankyrins are over‐represented (Foster et al., 2015). It is notable that this response takes time to develop. At 1 dai, none of the MycEx families are over‐represented, and at 4 dai only the RLP are over‐represented.

A search among gene lists related to arbuscular mycorrhization finds some MycEx genes, but they are not over‐represented in any set we examined (Bravo et al., 2016; Delaux et al., 2014; Recchia et al., 2018; Sugimura & Saito, 2017; Vangelisti et al., 2018) (Supporting Information Table S8).

## 4 DISCUSSION

### 4.1 MycEx gene families have a role in defense signaling

After demonstrating that the NBS gene family shows significant expansions in comparison of AM with NM species, and ECM with AM species, we conducted a search for other large gene families with similar properties. We found five additional families, which together with NBS we call the MycEx families. The five additional families are:

#### 4.1.1 Bulb‐type lectin receptor kinases (B‐type RKs, also called G‐type RKs)

This family includes the S‐locus receptor kinases that play a role in self‐incompatibility during fertilization (Takasaki et al., 2000), and the LORE receptor kinase that recognizes lipopolysaccharides from Gram‐negative bacteria (Ranf et al., 2015). A functional role in symbiosis has yet to be demonstrated, although Favre et al. (2014) identified three B‐type RKs in the conserved genetic module common to AM species.

#### 4.1.2 Glutamate Receptor‐Like (GLR)

These are the homologs of ionotropic glutamate receptors in mammals. Plant GLRs respond to a range of amino acids, and in most cases their functional roles remain to be clarified (Forde & Roberts, 2014). Some members of this family participate in long‐distance signaling and wound responses (Mousavi, Chauvin, Pascaud, Kellenberger, & Farmer, 2013), but a role in symbiotic competence has not been demonstrated. Notably, AM symbiosis can up‐regulate glutamate synthase in rice (Perez‐Tienda, Correa, Azcon‐Aguilar, & Ferrol, 2014), which suggests the possibility that glutamate participates in a long‐distance signal of mycorrhizal status to the rest of the plant.

#### 4.1.3 PGG‐Ankyrins

PGG is a domain of unknown function common to a large clade of transmembrane ankyrins. This family includes the gene ACCELERATED CELL DEATH 6 (ACD6), which plays a role in immunity and systemic acquired resistance (Rate, Cuenca, Bowman, Guttman, & Greenberg, 1999), and INEFFECTIVE GREENISH NODULES (IGN1), which regulates symbiosis with nitrogen‐fixing bacteria (Kumagai et al., 2007).

#### 4.1.4 Receptor‐Like Proteins (RLP)

Receptor‐like proteins are transmembrane proteins with an LRR domain that extends into the extracellular space, but they lack a kinase domain inside the cell. Their role in signaling is thus mediated by binding to other proteins on the cell surface. Some RLPs are known to play important roles in plant development, and others in immunity (Wang et al., 2008). Notably, many RLPs interact with SOBIR1/EVR to establish an immune response against fungal pathogens (Liebrand et al., 2013).

#### 4.1.5 Wall‐associated receptor kinases (WAK/WAKL)

Many WAKs have an extracellular domain that binds oligogalacturonides (OGs), products of cell wall damage. Consistent with this, WAKs have a role in both cell growth and pathogen response (Kohorn & Kohorn, 2012).

We found additional evidence for a connection between the MycEx families and innate immunity from immune response transcriptomes. While we did not attempt a systematic survey, we found five transcriptomes – four in *A. thaliana* and one in *P. tremuloides* ‐ where the majority of MycEx gene families were over‐represented in lists of genes up‐regulated in response to pathogen infection or immune elicitors. One notable feature of the *P. tremuloides* data is the MycEx gene response is detectable at 15 days after infection (dai), but not earlier (RLP being the exception, with over‐representation detectable at 4 dai).

The six MycEx gene families together comprise hundreds of loci per species. The median number of MycEx genes in NM, AM, and ECM plant species is 322, 636, and 1,657, respectively. Since NBS proteins are largely cytoplasmic while the others are mostly localized at the plasma membrane, we conclude that large segments of the plant defense repertoire expand in parallel with mycorrhizal competence.

### 4.2 MycEx gene families do not expand significantly with woodiness

It is notable that the MycEx gene family has substantial overlap with the list of gene families that expand in a comparison of woody vs nonwoody plant species previously published by Plomion et al. (2018) (see their Supplementary Dataset 7). Of the clades highlighted in their analysis, 5/15 are in the NBS family, 4/15 are B‐type receptor kinases, 2/15 are RLPs, and one clade is in the WAK family. The only large family highlighted by their analysis but absent in ours is the family of Leucine‐rich repeat receptor‐like kinases (LRR‐RLKs; 3/15 clades). Looking back to our own analysis, we find that LRR‐RLKs do expand significantly with mycorrhizal competence (category 1 in Supporting Information Table S4), but the size of the expansions falls short of our 50% threshold for inclusion in the MycEx list (46% from NM to AM and 48% from AM to ECM). Similarly, only two of the six MycEx families – GLR and PGG‐ankyrins – are absent from the Plomion et al. (2018) list.

The overlap between the MycEx family list and the woody expansion found by Plomion et al. (2018) is not surprising. The NM species on our list are nonwoody and the ECM species are woody, so the two characteristics are not independent. We conducted a simultaneous linear regression to quantify the relative importance of woodiness and mycorrhizal competence for the observed expansions in the MycEx families. This regression finds a larger correlation with mycorrhizal competence than with woodiness for all six families. More importantly, the regression coefficients for woodiness are not statistically different from 0, while those for mycorrhizal competence are significant. As an additional check, we tested for a dependence on woodiness just among the 27 AM species on our list. None was found.

As mentioned in the Introduction, there has been repeated speculation that plant defense gene families may expand in woody perennials as an adaptation for a long life‐span (see Tuskan et al., 2006; Yang et al., 2008; Plomion et al., 2018 and the opinion article Tobias & Guest, 2014). By contrast, our results suggest that the observed correlations of gene family size with woodiness may be an artifact of a stronger correlation with mycorrhizal competence. It should be emphasized, however, that our analysis relies on Pfam domain assignments (Punta et al., 2012), rather than the more highly resolved sub‐families used by Plomion et al. (2018), so it is possible that their clades have a stronger dependence on woodiness than found by us.

### 4.3 A hypothesis to explain MycEx family expansion

Our results suggest that the diversity of immune signaling components expands with mycorrhizal competence, but we have not yet considered possible explanations. In this section, we consider the hypothesis that this correlation is related to the species diversity of microbes in each kind of symbiotic association.

Arbuscular mycorrhiza fungi are monophyletic, and comprise ~150 species (Schussler, Schwarzott, & Walker, 2001). Field observation and transplant experiments suggest that most AM fungi are promiscuous, able to colonize any AM‐competent host plant (reviewed in Smith & Read, 2008). ECM fungal species are much more diverse than AM fungi. The ECM character has evolved independently in dozens of distinct fungal lineages, and includes thousands of species (Tedersoo & Brundrett, 2017; Tedersoo & Smith, 2013). ECM fungi typically form assemblages of 10 or more species that co‐occur on the roots of a single host plant. The composition of these assemblages varies across the geographic range of the host, so that a single ECM plant species may be competent to form symbioses with hundreds, or even thousands of distinct fungal species (Trappe, 1977; van der Linde et al., 2018; Van Geel et al., 2018). In addition, the ECM plant species we consider retain their AM competence (Figure 1; Supporting Information Table S1). Thus, nonmycorrhizal, arbuscular mycorrhizal, and ectomycorrhizal plant species lie on a continuum of symbiote diversity, NM < AM < ECM.

As discussed in the Introduction, there is a growing body of evidence that the plant immune system regulates interactions with both pathogenic and nonpathogenic microbes (see, e.g. Madsen et al., 2003; Yang et al., 2010; Miyata et al., 2014; and the reviews Cook et al., 2015; Hacquard et al., 2017). Consistent with this, our results suggest that the diversity of nonpathogenic microbes is a major driver of immune repertoire diversification. A host plant competent to form symbiotic associations with a greater diversity of symbiotic fungal species will need to rely on a greater diversity of signals to distinguish pathogenic from nonpathogenic fungi, especially in those cases where the symbiotic species is closely related to a pathogenic one. Therefore, the expansion of MycEx families may be an adaptation for symbiotic promiscuity.

### 4.4 Caveats

The symbiote diversity hypothesis outlined above offers a possible explanation for our results, but there are several caveats or potential objections we wish to consider.

First, genomic and transcriptomic approaches have mostly failed to find a significant relationship between the MycEx families and mycorrhizal symbiosis. Lists of genes conserved across AM plant species have relatively few MycEx genes (Delaux et al., 2014; Bravo et al., 2016; and Supporting Information Table S8). The only statistically significant over‐representation we found is six RLPs in the list compiled by Delaux et al. (2014). Similarly, transcriptomic studies of genes up‐regulated in response to AM colonization find some MycEx genes, but no family is over‐represented (Sugimura & Saito, 2017; Recchia et al., 2018; Vangelisti et al., 2018; and Supporting Information Table S8). Transcriptomic studies do report up‐regulation of defense‐related genes following AM colonization, but these genes are mostly downstream of the signaling pathways represented by the MycEx familes (Calabrese et al., 2017; Hohnjec, Vieweg, Puhler, Becker, & Kuster, 2005). We thus have the paradox that MycEx gene families do not appear to be especially active following AM colonization.

One possible explanation for this result comes from studies of legume‐rhizobia symbioses, which share many signaling pathways in common with mycorrhiza and have been more thoroughly studied (reviewed in Parniske, 2008). Transcriptomic studies of rhizobial symbiosis show an up‐regulation of plant defenses in response to the initial infection, followed by a suppression of those pathways after the successful establishment of symbiosis (Kouchi et al., 2004; Lohar et al., 2006; Maunoury et al., 2010). If the role of the innate immune system during AM colonization is similar to nodule formation, then any increase in MycEx family expression may be localized and transient, and therefore difficult to detect.

A second caveat to consider is that the AM and ECM fungi do not constitute the entire plant microbiome. Flowering plants also host large communities of bacterial and fungal endophytes – species that live within the plant without causing disease symptoms (reviewed in Porras‐Alfaro & Bayman, 2011; Reinhold‐Hurek, Bunger, Burbano, Sabale, & Hurek, 2015). The diversity of microbial endophytes is a focus of much current research, with studies finding about ~100 endophyte species per plant host (Lundberg et al., 2012). There is also a complex and poorly understood network of interactions between plant roots and the microbes that live at the root‐soil interface. The fact that AM and ECM symbioses stand out against this background is somewhat surprising.

Lastly, if the observed expansion of the MycEx gene families is principally to detect signals derived from invading microbes, that implies a much higher diversity of microbe‐originated signals than is currently known. One line of evidence consistent with the existence of additional signals comes from recent work sequencing the genomes of dozens of fungal species, both pathogenic and nonpathogenic (Kohler et al., 2015; Lo Presti et al., 2015). This reveals the presence of hundreds of short (<300 aa) proteins with secretion peptide signals and little similarity to known gene families. The extent to which these secreted proteins play a role in microbe‐host signaling remains to be explored.

Thus, while the observed correlation between mycorrhizal competence and immune repertoire size is strong, our proposed explanation in terms of symbiote species diversity remains speculative.

### 4.5 Comparisons with legume‐rhizobia symbiosis

We find that the NBS and other MycEx gene families do not expand significantly in the rhizobial legumes. This is consistent with our hypothesis about symbiote species diversity, since many legume species are competent to associate with fewer than ~10 species of rhizobia (reviewed in Dilworth et al., 2008). However, there are several confounding factors that need to be considered. First, since all the rhizobial species in our data set fall in a single family (Fabaceae, the legumes), we must be cautious about generalizing to rhizobia‐competent species outside the legumes. Second, the genomes of rhizobia display a high degree of genetic variation, and include evidence for the horizontal transfer of genes related to the establishment and maintenance of symbiosis (Epstein et al., 2012; Sugawara et al., 2013). Thus, the genetic diversity of rhizobia may not be well‐estimated by simple species counts. Third, there are important differences in the cell biology of rhizobial and mycorrhizal symbioses. Rhizobia occupy specialized root nodules that isolate them from the soil environment (reviewed in Dilworth et al., 2008; Guinel, 2009). This contrasts with fungal symbiosis, where the mycorrhizal filament network remains in direct contact with both the soil and the cells of the host plant root (reviewed in Smith & Read, 2008). Thus, the maintenance of mycorrhizal symbiosis may present a greater ongoing challenge to the plant immune system than rhizobial symbiosis.

## 5 CONCLUSION

In this paper, we have shown that the plant immune repertoire expands with mycorrhizal competence, and argued that symbiote diversity is a plausible explanation for this expansion. This idea has interesting parallels with the proposal that the evolution of the adaptive immune system in vertebrates facilitated the competence for a diverse and stable gut microbiota (McFall‐Ngai, 2007). The role of symbiote diversity as a driver of immune system expansion may be common to both kingdoms.

Some related work was recently published by Munch et al. (2018). They identified a clade of NBS genes with relatively few members in the Brassicaceae, and speculated that NM plants may have evolved immune signaling pathways distinct from the NBS system to compensate. We cannot rule out this explanation, but our observation that NM species have deficits in multiple defense pathways, not just NBS, makes this explanation less likely.

There are several ways to expand on the current paper that would improve confidence in our hypothesis. To better assess the significance of rhizobial symbiosis, it would be helpful to have multiple genomes from nonleguminous rhizobial species, and also from leguminous species that do not support rhizobial symbiosis. The hypothesis may be further tested using the genomes of plant species with distinct types of mycorrhizal association, such as orchids and the Ericaceae (reviewed in Smith & Read, 2008). To clarify the relative importance of pathogens, endophytes, and mycorrhizas for immune expansions, it would helpful to have a more complete survey of microbial diversity in all three classes. Another topic of interest is the search for sub‐families within the MycEx gene families that show especially strong dependence on mycorrhizal competence, or sub‐families that expanded during the evolutionary development of mycorrhiza (e.g. Yue, Meyers, Chen, Tian, & Yang, 2012). These projects may point the way to an improved functional understanding of the relationship between the immune repertoire and fungal symbiosis.

## CONFLICT OF INTEREST

The authors declare no competing interests.

## AUTHOR CONTRIBUTIONS

E.M.K. planned the research, conducted the statistical tests, and wrote the manuscript. S.A.S., H.J.Y. and J.W.C. contributed to the genome analysis. D.H.R.M. discussed the research and contributed to the manuscript.

## Data Availability

All data are available in the main text or the Supporting information Materials.
