# Supplementary material for: Flowering plant immune repertoires expand under mycorrhizal symbiosis
Source: Plant Direct. 2019 Mar 5;3(3):e00125. doi: 10.1002/pld3.125 (PMC6508770; doi:10.1002/pld3.125)
Supplement: Supplementary file 2 [file PLD3-3-e00125-s002.pdf]

Supplementary Materials for

Flowering plant immune repertoires expand under mycorrhizal symbiosis

Eric M. Kramer, Samantha A. Statter, Ho Jun Yi, Joseph W. Carlson, and Donald H. R. McClelland.

Correspondence to: ekramer@simons-rock.edu

This PDF file includes:

Legends for Supplemental Tables

References for Supplemental Files

Legends for Supplemental Tables

Table S1. Details and references for 39 species of angiosperms used in this paper.

Table S2. Gene family size. Total number of loci in each of the 331 gene families considered in this paper. Three curated gene families are also included in the list: Leucine-rich repeat receptor-like kinases (LRR-RLK), soluble kinases, and receptor-like proteins (RLP). Pfam domains were identified using Pfamscan (Punta *et al.* 2012) and transmembrane domains were identified using Phobius (Kall *et al.* 2007).

Table S3. Regression results. See the Methods section for details of the calculation. The b_w and b_{myc} are regression coefficients for woodiness and mycorrhizal competence respectively. Also shown is the probability p that the b-value is different from 0, and the cumulative false discovery rate (q) estimated using the technique of Benjamini & Hochberg (1995) for a list with six members.

Table S4. Comparisons of gene family size for plant species with different mycorrhizal competence. The same 331 gene families from Table S2, showing relevant statistical comparisons. The p-values for significant differences are calculated using the exact, one-sided Wilcoxon-Mann-Whitney rank sum test, implemented using the COIN package in R (COIN version 1.2-2, (Hothorn *et al.* 2006); R version 3.5.1 (Team 2018)). Cumulative false discovery rate (q) estimated using the technique of Benjamini & Hochberg (1995) for a list with 331 members. Columns I to M show median family sizes and their ratios. The category labels are assigned as follows: category 1 families are significantly larger in AM than NM, and significantly larger in ECM than AM (difference is significant if false discovery rate $q < .05$). Category 2 families are significantly larger in AM than NM only. Category 3 families are significantly larger in ECM than AM only. Category 4 families have no significant differences.

Table S5. Size of the Mycorrhizal-Expanded (MycEx) gene families in 39 angiosperm species. Pfam domains were identified using Pfamscan (Punta et al. 2012) and transmembrane domains were identified using Phobius (Kall et al. 2007).

Table S6. Measures of the MycEx gene families. Columns B through F show median counts per species in NM, AM, and ECM categories, and ratios of same. Columns H through X show significance tests of NM < AM, AM < ECM, NM<ECM, (nonwoody AM) < (woody AM), and (non-leguminous AM) < (leguminous AM). The p-values are calculated using the exact, one-sided Wilcoxon-Mann-Whitney rank sum test, implemented using the COIN package in R (COIN version 1.2-2, (Hothorn et al. 2006); R version 3.5.1 (Team 2018)) and cumulative false discovery rates (*q*) are estimated using the technique of Benjamini & Hochberg (1995) for a starting set of 331 gene families.

Table S7. Significance of the MycEx gene families in immune response transcriptomes. Columns show the total number of loci from each family in the gene set listed at top, and the p-value for over-representation using a one-sided hypergeometric test, implemented in Excel (v. 16, Microsoft). Refs. (Denoux *et al.* 2008; Wan *et al.* 2008; Foster *et al.* 2015; Bernsdorff *et al.* 2016) are discussed further in the Methods section. Cumulative false discovery rates (*q*) are estimated using the technique of Benjamini & Hochberg (1995) for a list of 6 values. Gray cells indicate entries not significantly over-represented.

Table S8. Counts of MycEx family members in gene sets related to AM symbiosis. Delaux et al. (Delaux *et al.* 2014) and Bravo et al. (Bravo *et al.* 2016) used genomic and transcriptomic approaches to identify genes conserved across AM species. Refs. (Sugimura & Saito 2017; Recchia *et al.* 2018; Vangelisti *et al.* 2018) found genes upregulated in the transcriptomes of AM-inoculated plants in *Solanum lycopersicum*, *Phaseolus vulgaris*, and *Helianthus annuus* respectively. The probability *p* of over-representation is determined using the one-sided hypergeometric test (Excel, v. 16, Microsoft). The orange cell highlights the only $p < .05$.

References for Supplemental Files

Alguacil, M. d. M., E. Torrecillas, Z. Lozano and A. Roldan (2011). Evidence of Differences between the Communities of Arbuscular Mycorrhizal Fungi Colonizing Galls and Roots of Prunus persica Infected by the Root-Knot Nematode Meloidogyne incognita. *Applied And Environmental Microbiology* 77: 8656-8661.

Badouin, H., J. Gouzy, C. J. Grassa, F. Murat, S. E. Staton, L. Cottret, C. Lelandais-Briere, G. L. Owens, S. Carrere, B. Mayjonade, et al. (2017). The sunflower genome provides insights into oil metabolism, flowering and Asterid evolution. *Nature* 546(7656): 148-152.

Beier, S., A. Himmelbach, C. Colmsee, X. Q. Zhang, R. A. Barrero, Q. Zhang, L. Li, M. Bayer, D. Bolser, S. Taudien, et al. (2017). Construction of a map-based reference genome sequence for barley, Hordeum vulgare L. *Sci Data* 4: 170044.

Benjamini, Y. and Y. Hochberg (1995). Controlling the false discovery rate: a practical and powerful approach to multiple testing. *Journal of the Royal Statistical Society. Series B* 57: 289-300.

Bernsdorff, F., A. C. Doring, K. Gruner, S. Schuck, A. Brautigam and J. Zeier (2016). Pipecolic Acid Orchestrates Plant Systemic Acquired Resistance and Defense Priming via Salicylic Acid-Dependent and -Independent Pathways. *Plant Cell* 28(1): 102-129.

Bravo, A., T. York, N. Pumplin, L. A. Mueller and M. J. Harrison (2016). Genes conserved for arbuscular mycorrhizal symbiosis identified through phylogenomics. *Nat Plants* 2: 15208.

Bredeson, J. V., J. B. Lyons, S. E. Prochnik, G. A. Wu, C. M. Ha, E. Edsinger-Gonzales, J. Grimwood, J. Schmutz, I. Y. Rabbi, C. Egesi, et al. (2016). Sequencing wild and cultivated cassava and related species reveals extensive interspecific hybridization and genetic diversity. *Nat Biotechnol* 34(5): 562-570.

Brundrett, M. C. (2009). Mycorrhizal associations and other means of nutrition of vascular plants: understanding the global diversity of host plants by resolving conflicting information and developing reliable means of diagnosis. *Plant Soil* 320: 37-77.

Clouse, J. W., D. Adhikary, J. T. Page, T. Ramaraj, M. K. Deyholos, J. A. Udall, D. J. Fairbanks, E. N. Jellen and P. J. Maughan (2016). The Amaranth Genome: Genome, Transcriptome, and Physical Map Assembly. *Plant Genome* 9(1).

De Vega, J. J., S. Ayling, M. Hegarty, D. Kudrna, J. L. Goicoechea, A. Ergon, O. A. Rognli, C. Jones, M. Swain, R. Geurts, et al. (2015). Red clover (Trifolium pratense L.) draft genome provides a platform for trait improvement. *Sci Rep* 5: 17394.

Delaux, P. M., K. Varala, P. P. Edger, G. M. Coruzzi, J. C. Pires and J. M. Ane (2014). Comparative phylogenomics uncovers the impact of symbiotic associations on host genome evolution. *PLoS Genet* 10(7): e1004487.

Denoux, C., R. Galletti, N. Mammarella, S. Gopalan, D. Werck, G. De Lorenzo, S. Ferrari, F. M. Ausubel and J. Dewdney (2008). Activation of defense response pathways by OGs and Flg22 elicitors in Arabidopsis seedlings. *Mol Plant* 1(3): 423-445.

Dickie, I. A., R. T. Koide and A. C. Fayish (2001). Vesicular–arbuscular mycorrhizal infection of *Quercus rubra* seedlings. *New Phytologist* 151: 257-264.

Dohm, J. C., A. E. Minoche, D. Holtgrawe, S. Capella-Gutierrez, F. Zakrzewski, H. Tafer, O. Rupp, T. R. Sorensen, R. Stracke, R. Reinhardt, et al. (2014). The genome of the recently domesticated crop plant sugar beet (Beta vulgaris). *Nature* 505(7484): 546-549.

Droc, G., D. Lariviere, V. Guignon, N. Yahiaoui, D. This, O. Garsmeur, A. Dereeper, C. Hamelin, X. Argout, J. F. Dufayard, et al. (2013). The banana genome hub. *Database (Oxford)* 2013: bat035.

Foster, A. J., G. Pelletier, P. Tanguay and A. Seguin (2015). Transcriptome Analysis of Poplar during Leaf Spot Infection with Sphaerulina spp. *PLoS One* 10(9): e0138162.

Goodstein, D. M., S. Shu, R. Howson, R. Neupane, R. D. Hayes, J. Fazo, T. Mitros, W. Dirks, U. Hellsten, N. Putnam, et al. (2012). Phytozome: a comparative platform for green plant genomics. *Nucleic Acids Research* 40: D1178-D1186.

Groth, M., S. Kosuta, C. Gutjahr, K. Haage, S. L. Hardel, M. Schaub, A. Brachmann, S. Sato, S. Tabata, K. Findlay, et al. (2013). Two Lotus japonicus symbiosis mutants impaired at distinct steps of arbuscule development. *Plant J* 75(1): 117-129.

Hellsten, U., K. M. Wright, J. Jenkins, S. Shu, Y. Yuan, S. R. Wessler, J. Schmutz, J. H. Willis and D. S. Rokhsar (2013). Fine-scale variation in meiotic recombination in Mimulus inferred from population shotgun sequencing. *Proc Natl Acad Sci U S A* 110(48): 19478-19482.

Hirsch, C. N., C. D. Hirsch, A. B. Brohammer, M. J. Bowman, I. Soifer, O. Barad, D. Shem-Tov, K. Baruch, F. Lu, A. G. Hernandez, et al. (2016). Draft Assembly of Elite Inbred Line

PH207 Provides Insights into Genomic and Transcriptome Diversity in Maize. *Plant Cell* 28(11): 2700-2714.

Hohnjec, N., M. F. Vieweg, A. Puhler, A. Becker and H. Kuster (2005). Overlaps in the transcriptional profiles of Medicago truncatula roots inoculated with two different Glomus fungi provide insights into the genetic program activated during arbuscular mycorrhiza. *Plant Physiol* 137(4): 1283-1301.

Hong, J. J., Y.-S. Park, A. Bravo, K. K. Bhattarai, D. A. Daniels and M. J. Harrison (2012). Diversity of morphology and function in arbuscular mycorrhizal symbioses in *Brachypodium distachyon*. *Planta* 236: 851-865.

Hothorn, T., K. Hornik, M. A. van de Wiel and A. Zeileis (2006). coin: A Computational Framework for Conditional Inference, R package version 0.4-5, http://CRAN.R-project.org/

Initiative, I. P. G., I. Verde, A. G. Abbott, S. Scalabrin, S. Jung, S. Shu, F. Marroni, T. Zhebentyayeva, M. T. Dettori, J. Grimwood, et al. (2013). The high-quality draft genome of peach (Prunus persica) identifies unique patterns of genetic diversity, domestication and genome evolution. *Nat Genet* 45(5): 487-494.

International Brachypodium, I. (2010). Genome sequencing and analysis of the model grass Brachypodium distachyon. *Nature* 463(7282): 763-768.

Iorizzo, M., S. Ellison, D. Senalik, P. Zeng, P. Satapoomin, J. Huang, M. Bowman, M. Iovene, W. Sanseverino, P. Cavagnaro, et al. (2016). A high-quality carrot genome assembly provides new insights into carotenoid accumulation and asterid genome evolution. *Nat Genet* 48(6): 657-666.

Jaillon, O., J. M. Aury, B. Noel, A. Policriti, C. Clepet, A. Casagrande, N. Choisne, S. Aubourg, N. Vitulo, C. Jubin, et al. (2007). The grapevine genome sequence suggests ancestral hexaploidization in major angiosperm phyla. *Nature* 449(7161): 463-467.

Kall, L., A. Krogh and E. L. Sonnhammer (2007). Advantages of combined transmembrane topology and signal peptide prediction--the Phobius web server. *Nucleic Acids Res* 35(Web Server issue): W429-432.

Lamesch, P., T. Z. Berardini, D. Li, D. Swarbreck, C. Wilks, R. Sasidharan, R. Muller, K. Dreher, D. L. Alexander, M. Garcia-Hernandez, et al. (2012). The Arabidopsis

Information Resource (TAIR): improved gene annotation and new tools. *Nucleic Acids Res* 40(Database issue): D1202-1210.

Liu, S., Y. Liu, X. Yang, C. Tong, D. Edwards, I. A. Parkin, M. Zhao, J. Ma, J. Yu, S. Huang, et al. (2014). The Brassica oleracea genome reveals the asymmetrical evolution of polyploid genomes. *Nat Commun* 5: 3930.

McCormick, R. F., S. K. Truong, A. Sreedasyam, J. Jenkins, S. Shu, D. Sims, M. Kennedy, M. Amirebrahimi, B. D. Weers, B. McKinley, et al. (2018). The Sorghum bicolor reference genome: improved assembly, gene annotations, a transcriptome atlas, and signatures of genome organization. *Plant J* 93(2): 338-354.

Menge, J. A., E. L. V. Johnson and R. G. Platt (1978). Mycorrhizal dependency of several *Citrus* cultivars under three nutrient regimes. *New Phytologist* 81: 553-559.

Ming, R., R. VanBuren, C. M. Wai, H. Tang, M. C. Schatz, J. E. Bowers, E. Lyons, M. L. Wang, J. Chen, E. Biggers, et al. (2015). The pineapple genome and the evolution of CAM photosynthesis. *Nat Genet* 47(12): 1435-1442.

Mishra, B., D. K. Gupta, M. Pfenninger, T. Hickler, E. Langer, B. Nam, J. Paule, R. Sharma, B. Ulaszewski, J. Warmbier, et al. (2018). A reference genome of the European beech (Fagus sylvatica L.). *Gigascience* 7(6).

Motamayor, J. C., K. Mockaitis, J. Schmutz, N. Haiminen, D. Livingstone, 3rd, O. Cornejo, S. D. Findley, P. Zheng, F. Utro, S. Royaert, et al. (2013). The genome sequence of the most widely cultivated cacao type and its use to identify candidate genes regulating pod color. *Genome Biol* 14(6): r53.

Myburg, A. A., D. Grattapaglia, G. A. Tuskan, U. Hellsten, R. D. Hayes, J. Grimwood, J. Jenkins, E. Lindquist, H. Tice, D. Bauer, et al. (2014). The genome of Eucalyptus grandis. *Nature* 510(7505): 356-362.

Ngosong, C., M. Jarosch, J. Raupp, E. Neumann and L. Ruess (2010). The impact of farming practice on soil microorganisms and arbuscular mycorrhizal fungi: Crop type versus long-term mineral and organic fertilization. *Applied Soil Ecology* 46: 134-142.

Nielsen, S. L., I. Thingstrup and C. Wigand (1999). Apparent lack of vesicular-arbuscular mycorrhiza (VAM) in the seagrasses Zostera marina L. and Thalassia testudinum Banks ex Konig. *Aquatic Botany* 63: 261-266.

Olsen, J. L., P. Rouze, B. Verhelst, Y. C. Lin, T. Bayer, J. Collen, E. Dattolo, E. De Paoli, S. Dittami, F. Maumus, et al. (2016). The genome of the seagrass Zostera marina reveals angiosperm adaptation to the sea. *Nature* 530(7590): 331-335.

Ouyang, S., W. Zhu, J. Hamilton, H. Lin, M. Campbell, K. Childs, F. Thibaud-Nissen, R. L. Malek, Y. Lee, L. Zheng, et al. (2007). The TIGR Rice Genome Annotation Resource: improvements and new features. *Nucleic Acids Res* 35(Database issue): D883-887.

Plomion, C., J. M. Aury, J. Amselem, T. Leroy, F. Murat, S. Duplessis, S. Faye, N. Francillonne, K. Labadie, G. Le Provost, et al. (2018). Oak genome reveals facets of long lifespan. *Nat Plants* 4(7): 440-452.

Punta, M., P. C. Coggill, R. Y. Eberhardt, J. Mistry, J. Tate, C. Boursnell, N. Pang, K. Forslund, G. Ceric, J. Clements, et al. (2012). The Pfam protein families database. *Nucleic Acids Research* 40: D290-D301.

Recchia, G. H., E. R. Konzen, F. Cassieri, D. G. G. Caldas and S. M. Tsai (2018). Arbuscular Mycorrhizal Symbiosis Leads to Differential Regulation of Drought-Responsive Genes in Tissue-Specific Root Cells of Common Bean. *Front Microbiol* 9: 1339.

Reyes-Chin-Wo, S., Z. Wang, X. Yang, A. Kozik, S. Arikit, C. Song, L. Xia, L. Froenicke, D. O. Lavelle, M. J. Truco, et al. (2017). Genome assembly with in vitro proximity ligation data and whole-genome triplication in lettuce. *Nat Commun* 8: 14953.

Sato, S., Y. Nakamura, T. Kaneko, E. Asamizu, T. Kato, M. Nakao, S. Sasamoto, A. Watanabe, A. Ono, K. Kawashima, et al. (2008). Genome structure of the legume, Lotus japonicus. *DNA Res* 15(4): 227-239.

Schmutz, J., S. B. Cannon, J. Schlueter, J. Ma, T. Mitros, W. Nelson, D. L. Hyten, Q. Song, J. J. Thelen, J. Cheng, et al. (2010). Genome sequence of the palaeopolyploid soybean. *Nature* 463(7278): 178-183.

Schmutz, J., P. E. McClean, S. Mamidi, G. A. Wu, S. B. Cannon, J. Grimwood, J. Jenkins, S. Shu, Q. Song, C. Chavarro, et al. (2014). A reference genome for common bean and genome-wide analysis of dual domestications. *Nat Genet* 46(7): 707-713.

Slotte, T., K. M. Hazzouri, J. A. Agren, D. Koenig, F. Maumus, Y. L. Guo, K. Steige, A. E. Platts, J. S. Escobar, L. K. Newman, et al. (2013). The Capsella rubella genome and the genomic consequences of rapid mating system evolution. *Nat Genet* 45(7): 831-835.

Sugimura, Y. and K. Saito (2017). Comparative transcriptome analysis between *Solanum lycopersicum* L. and *Lotus japonicus* L. during arbuscular mycorrhizal development. *Soil Science and Plant Nutrition* 63: 127-136.

Tang, H., V. Krishnakumar, S. Bidwell, B. Rosen, A. Chan, S. Zhou, L. Gentzbittel, K. L. Childs, M. Yandell, H. Gundlach, et al. (2014). An improved genome release (version Mt4.0) for the model legume Medicago truncatula. *BMC Genomics* 15: 312.

Team, R. C. (2018). R: A language and environment for statistical computing. Vienna, Austria, R Foundation for Statistical Computing.

Tomato Genome, C. (2012). The tomato genome sequence provides insights into fleshy fruit evolution. *Nature* 485(7400): 635-641.

Tuskan, G. A., S. Difazio, S. Jansson, J. Bohlmann, I. Grigoriev, U. Hellsten, N. Putnam, S. Ralph, S. Rombauts, A. Salamov, et al. (2006). The genome of black cottonwood, Populus trichocarpa (Torr. & Gray). *Science* 313(5793): 1596-1604.

Unver, T., Z. Wu, L. Sterck, M. Turktas, R. Lohaus, Z. Li, M. Yang, L. He, T. Deng, F. J. Escalante, et al. (2017). Genome of wild olive and the evolution of oil biosynthesis. *Proc Natl Acad Sci U S A* 114(44): E9413-E9422.

Vangelisti, A., L. Natali, R. Bernardi, C. Sbrana, A. Turrini, K. Hassani-Pak, D. Hughes, A. Cavallini, M. Giovannetti and T. Giordani (2018). Transcriptome changes induced by arbuscular mycorrhizal fungi in sunflower (Helianthus annuus L.) roots. *Scientific Reports* 8: 4.

Varshney, R. K., C. Song, R. K. Saxena, S. Azam, S. Yu, A. G. Sharpe, S. Cannon, J. Baek, B. D. Rosen, B. Tar'an, et al. (2013). Draft genome sequence of chickpea (Cicer arietinum) provides a resource for trait improvement. *Nat Biotechnol* 31(3): 240-246.

Velasco, R., A. Zharkikh, J. Affourtit, A. Dhingra, A. Cestaro, A. Kalyanaraman, P. Fontana, S. K. Bhatnagar, M. Troggio, D. Pruss, et al. (2010). The genome of the domesticated apple (Malus x domestica Borkh.). *Nat Genet* 42(10): 833-839.

Wan, J., X.-C. Zhang, D. Neece, K. M. Ramonell, S. Clough, S.-y. Kim, M. G. Stacey and G. Stacey (2008). A LysM Receptor-Like Kinase Plays a Critical Role in Chitin Signaling and Fungal Resistance in Arabidopsis. *Plant Cell* 20: 471-481.

Wang, B. and Y. L. Qiu (2006). Phylogenetic distribution and evolution of mycorrhizas in land plants. *Mycorrhiza* 16(5): 299-363.

Wang, W., G. Haberer, H. Gundlach, C. Glasser, T. Nussbaumer, M. C. Luo, A. Lomsadze, M. Borodovsky, R. A. Kerstetter, J. Shanklin, et al. (2014). The Spirodela polyrhiza genome reveals insights into its neotenous reduction fast growth and aquatic lifestyle. *Nat Commun* 5: 3311.

Wang, Z., N. Hobson, L. Galindo, S. Zhu, D. Shi, J. McDill, L. Yang, S. Hawkins, G. Neutelings, R. Datla, et al. (2012). The genome of flax (Linum usitatissimum) assembled de novo from short shotgun sequence reads. *Plant J* 72(3): 461-473.

Wu, G. A., S. Prochnik, J. Jenkins, J. Salse, U. Hellsten, F. Murat, X. Perrier, M. Ruiz, S. Scalabrin, J. Terol, et al. (2014). Sequencing of diverse mandarin, pummelo and orange genomes reveals complex history of admixture during citrus domestication. *Nat Biotechnol* 32(7): 656-662.
