# Supplementary material for: Flowering plant immune repertoires expand under mycorrhizal symbiosis
Source: Plant Direct. 2019 Mar 5;3(3):e00125. doi: 10.1002/pld3.125 (PMC6508770; doi:10.1002/pld3.125)
Supplement: Supplementary file 3 [file PLD3-3-e00125-s003.pdf]

To the editor and reviewers,

In the new version I have tried to address all remaining reviewer comments, as detailed below.

Reviewer #1:

*My only suggestion to the authors is to carefully revise the text after the formatting of the new references, many of which appear in the text as with a double parenthesis as in "reviewed in (xxxx))".

A: changes made.

Reviewer #2:

*p.8 l.219: The authors compare ECM to NM and AM, but only give one p-value. Since these are two comparisons, two p-values should be given.

A: change made.

*p.8 l.224 & 226: please give the exact p-values.

A: change made.

*p.8 l.233 & 239: please add the exact p-values to the text.

A: change made.

*p.8/9 l.245-250: In this section it is evident that it was moved from M&M to results. It stands out. Therefore, this moved text does not yet read like a results section. Additional information should be added in (i.e. how many Pfam domains were initially querried). Was each Pfam domain counted as one large gene family? Why was it necessary to currate the LRR-RLK, RLPs and soluble kinases. I assume this is because they would occur partially in the same Pfam domains and then would also appear in additional pfam domains. Why was this done specifically for those three families and not for others? Also it seems similar issues arise with other families including the six families that the manuscript was focused on. This should be mentioned at this point. Also Table S2 should be mentioned when needed, i.e. in l.246 and l.249 while the sentence in l.249/250 should be deleted.

A: we have completely rewritten this paragraph.

*p.14 l.408: Do the authors mean AM plant or AM fungal species? Please clarify.

A: inserted the word “plant”

*p.15 l.440-444: Connection to the previous idea and the overall conclusion is not clear. Please clarify.

A: We have rewritten the last half of this paragraph.

*Table S2: There are some fields that have a Pfam domain number but the category field is empty. Please fix this.

A: labels added.

*p.1 l.25 "immune repertoire size" not "immune size"

A: change made.

*p.6 l.172: should be "... used an Affymetrix ..."

A: change made.
